# On the Disinfection of Air

**Published:** 1873-05

**Authors:** A. Ernest Sansom

**Affiliations:** M. D., Lond., M. R. C. P., Physician to the Royal Hospital for Diseases of the Chest, and to the Northeastern Hospital for Children


*On the Disinfection of Air.* By A. ERNEST SANSOM, M.D., Lond., M.R.C.P., Physician to the Royal Hospital for Diseases of the Chest, and to the Northeastern Hospital for Children.

Few physicians will be inclined to deny that the material poisons of many of the spreading diseases are capable of being wafted by the air from place to place and from person to person, and that in this manner the maladies of infection are in many instances transmitted. In certain diseases—among which may be especially enumerated variola, scarlatina, rubeola, pertussis, pyæmia, erysipelas, and diphtheria—the subjects are centres whence the evolution of disease-inducing particles takes place. It is obvious that complete isolation of an individual, and removal of all the chance of his directly infecting others, are practically impossible; and, even if such were possible, the disease-poison would yet be cast off to attach itself to any surrounding material, still capable of inducing its effects in the future. It would be a measure of the highest importance if the disease-poison, while existing in, or wafted by, the air, could be rendered inert. This is what I mean by disinfection of the air—the rendering inert of the disease-poison which in some infectious disorders is contained in, and transmitted by, the atmosphere.

Many practitioners have attached an importance to the attempt to disinfect the air poisoned by the emanations of those sick of an infectious disease, as a positive duty; and have adopted various methods of carrying it out. Others, however, urge that all attempts in this direction are futile—that it is impossible to disinfect the air.

Let me discuss briefly what is the nature of the material, which I have for convenience called *poison*, that we desire to render inert. It is ponderable, and obeys physical laws. It often remains active for long periods of time, and hence is unaffected by the frequent mutations of the atmosphere; but is so minute as to be entirely undemonstrable by any ordinary direct physical means, and undetectable by the highest powers of the microscope. The diffusion experiments of Chauveau and Sanderson have given convincing proof that in vaccinia, variola, and sheep-pox, the poison is a *solid*, insoluble and indiffusible. This conceded, the area of speculation becomes narrowed; for it is impossible to imagine the material an agent merely organic and gifted only with the properties of inanimate matter. If it be an organic solid, neither soluble nor diffusible, how does it interpenetrate the tissues of the body it so profoundly affects? and how can we explain its immeasurable powers of reproduction? It is not my purpose to trace the analogies of the disease-poison, nor follow the evidence which points to the probability that it is to be endowed with the properties of *living* matter. We must admit one of two views—that it is vitalized, or else it acts by an inexplicable process of catalysis; and if the analogy of other so-called catalytic processes be invoked for the elucidation, we shall find that, as science progresses, these become less and less explicable, on the hypothesis of causation by dead organic material in any state whatever. In fact, I believe it to be true, in the present state of science, that no kind of catalytic change of organic bodies ever takes place without the intervention of living things or their active secretions. I will now turn to the consideration of the methods which have been hitherto adopted for the disinfection of air, and the *rationale* of their action.

First come what are called OXIDIZING DISINFECTANTS.

1. Foremost among these are the alkaline permanganates, which, when dissolved in water, it is recommended to expose in open vessels to the air of a sick-room, or to diffuse in the form of spray throughout the atmosphere. The theory is, that these yield up oxygen to the disease-poison, and, altering its chemical constitution, impair its morbific properties. Now it must be remembered, in the first place, that these agents are entirely non-volatile. There can be, therefore, no commingling with the air. The particular portions of air which happen to come into immediate contact with the surface of the solution, no doubt have their contained organic particles oxidized; but how feeble a proportion do these bear to the atmosphere of an apartment! The nauseous products of putrefaction, the permanganates have a remarkable power of oxidizing, and thus rendering inodorous; they are excellent *deodorants;* but I have shown them to occupy a very low place both as arresting putrefaction and as killing the low forms of life. A little consideration must show that agents which operate by oxidation only can have but little effect upon the specific poisons of disease. Imagine the minute particles of disease-poison as it is after a long continued relaxation with large volumes of air, this air frequently containing its special oxidizing constituent—

ozone; or imagine it commingled with vast bulks of water; surely here are enough conditions for its speedy oxidation and destruction, if it be capable thereof. How insignificant must be the superadded effect of the oxidizing permanganate! In whatever bulk employed, the permanganates do not destroy the microzymes inducing putrefaction, existing in the air superincumbent upon them. From my view, therefore, of the nature of disease-poison, I am justified in concluding that the employment of permanganates as means for the disinfection of air is at once frivolous and futile.

2. Chlorine is also an oxidizing disinfectant; but this stands on higher ground as an air-purifier, because its volatility allows it to come in immediate contact with the particles suspended in the air. It is of a very high value as a deodorant; but its action on fermentation, putrefaction, and on the life of a low organism, is but feeble. I have seen fungi living luxuriantly in and amongst the particles of chloride of lime.

3. Iodine is much more efficacious. It is an oxidizing agent, and hence a deodorant; but it is more—it destroys microzymes, and arrests decomposition. It is of very easy application. If placed in open vessels about a room, it evaporates spontaneously, and is a very useful agent for disinfection of air.

4. Sulphurous acid has also a double action; it is a deodorizer, and hence a deodorant; and it is extremely powerful in destroying the life of low organisms. It has been used (the method of its evolution being merely burning brimstone from a metal plate), and, as the records seem to show, with much advantage.

5. Carbolic acid is a volatile agent, easily diffused throughout the air. It exerts no chemical action thereupon, and thus is not a deodorant; but in my opinion it holds the highest place as a disinfectant proper, for it kills the low forms of life existing in the atmosphere, even when it is present in but small proportion.

Starting with the view that the poisons of spreading diseases are minute particles of living matter, I will now turn to some experimental evidence bearing upon the question of the efficacy, positive and relative, of air-disinfectants. Observation of the effects of different agents, when commingled with the air, upon the low organisms met with in putrefactions, must for the most part offer *a fortiori* evidence; for in many infective liquids the presence of the minutest bacteria cannot be detected. Dr. Sanderson has, however, observed bacteria as the characteristic inhabitants of the infective fluids which transmit pyæmia, "and therefore probably the carriers of infection." Without assuming, however, anything further than that the poisons are *living*, we can, I think, obtain valuable analogical evidence; for the influences which kill bacteria, monads, and germs of fungi, could surely with great probability kill also the minuter particles of living matter constituting the infecting poison.

Experiment I, *showing that Bacteria and Monads are killed by Air which contains Carbolic Acid.*—A maceration of hard-boiled egg in cold water was observed on the third day after preparation. Multitudes of cylindrical and filiform bacteria and ovoid monads were observed in active movement (magnifying power, 500 and 750 diameters linear). A small drop was placed on a slide, and held for thirty seconds over the open mouth of a flask which contained a little carbolic acid. This was compared with another drop uninfluenced. At first, movements in each case were equally active. In about two minutes in the former, movements became more languid, and soon ceased in many instances. Some, however, continued perfectly active. Experiment was repeated with exposure to carbolic acid for one minute. Monads, at first active, began to move in smaller circles, then merely to revolve on their axes, and finally manifested merely a peculiar vibration. A very marked difference was shown between the exposed and the test-drops; in the former, a large number of bacteria were perfectly motionless. Experiment repeated with exposure for three and five minutes respectively: in the latter case, nearly all were dead. It was evident that the superficial portion was so affected as to render motionless all the bacteria; but in the deeper layer some, though comparatively a very small number, manifested active movement.

Observation has shown me that, whilst the effect of carbolized air upon a fluid capable of putrefaction is to prevent the formation of the usually dense follicle formed by dead bacteria, and to kill those which are superficial, it has no influence upon those below the surface level, which continue to manifest active movement.

Experiment II, *showing that the Germs in the Air, which develop into Fungi, are killed by Air which contains Carbolic Acid.*—Two precisely similar glass flasks were taken, and into each was poured some well boiled paste made from wheat flour. Each was provided with a cork perforated and fitted with tubes for the entrance and exit of air. Over the surface of the paste in A, 500 cubic feet of air were blown by means of a bellows, and then the flask was closed with India rubber tissue. B was treated precisely similar, except that the air first traversed the surface of six drachms of liquefied carbolic acid contained in another small flask. B was subsequently sealed. The loss of the liquid carbolic acid was half a fluid drachm. The flasks were then kept in identical conditions. In flask A, after a few days, little islands of mildew appeared; the paste fermented, and disengaged gas-bubbles; and now (thirteenth day) green mould covers the sides, and large masses of white and green mould are on the surface of the paste. In B the paste remains perfectly unchanged, and now (thirteenth day) presents not a trace of fungoid manifestation. These flasks were exhibited to the meeting, and it was manifest that, although more than three weeks had elapsed since their preparation, the paste over which carbolized air had been passed, remained without any sign of decomposition; the other presented a mass of mildew.

I have formerly shown that the appearance of penicillium and aspergillus can be entirely prevented by the presence of carbolic acid in the air supplied to the soil.

Experiment III, *showing that, whilst the presence of Carbolic Acid or Sulphurous Acid in the Air prevents Putrefaction and the concurrent Development of Living Forms, the so-called Oxidizing Disinfectants fail to prevent either.*—Four small wide-mouthed glass bottles, of similar shape and size, were filled with small cubes cut from a hard-boiled egg. They were then placed each in a small trough, into which was poured the following disinfectant agent in each case : A, commercial permanganate solution (red,) 1½ drachm; B, commercial chloride of aluminium, 1½ drachm; C, pharmacopœial sulphurous acid, 1 drachm; D, crude carbolic acid, 11 drops. Each was enclosed under a glass cover, so as to allow free communication of air both with the open bottle and the free surface of the disinfectant agent; but the cover itself was imbedded in cement, so as to exclude external air. At the end of seven days, the covers were removed, and the bottles containing the pieces of egg were each filled up with distilled water which had been well boiled and then allowed to cool. The covers were then reapplied, and the contents were examined three days afterwards. A (permanganate) was extremely fœtid, so turbid as to be absolutely opaque, and with a thick pellicle covering its surface. It teemed with small bacteria, and presented many fungoid cells; and the portions of egg were disintegrated; they were in a state of advanced decomposition. B (chloride of aluminium): very fœtid and turbid, rather less so than A. Every drop examined swarmed with small monads and bacteria. The egg-cubes were broken up, and presented signs of advanced decomposition. C (sulphurous acid): Fluid presented no fœtor; was perfectly transparent. The cubes of egg appeared to preserve their shape and condition exactly as when first introduced. On microscopic examination, many fields presented no trace of motile organism; but occasionally a few active bacteria were met with. D (carbolic acid): Fluid appeared almost clear, and the egg-cubes distinct. Very few bacteria presented themselves, usually five or six in a field; they were of unusual length. Decomposition was almost, but not entirely arrested.

The evidence which I have adduced, I think, fully shows that the presence of certain volatile or gaseous antiseptic agents in the air is sufficient to poison the lowest forms of organisms, to prevent the appearance and development of fungi, and to arrest the putrefaction of organic material. I hope it will be allowed that we who counsel the employment of gaseous or volatile antiseptics for the disinfection of air, do so with the clear understanding of a purpose to fulfill, and a train of analogical evidence that the means are adequate to the end. We believe the subtile poisons of disease to be minute particles of living matter; and that these, given off from a diseased person, can be contained in and wafted by the air. We do not contend that they are equally numerous with the harmless germs of living things which the air normally contains; but they are with them and among them. We show that the presence of antiseptic agents in the air destroys the harmless germs; and we contend that it is probable that it also destroys the disease-germs, which have strong analogical relations with the former, which certainly do not exceed them in bulk, and are probably far more minute than those which are obviously destroyed. As regards the nature of an efficient air-disinfectant, we find that it must be volatile or gaseous, and therefore capable of equable diffusion throughout the air. The non-volatile agents are inert. It is not the action of a chemical agent upon an organic chemical compound which constitutes disinfection, according to our view; but the action of a diffused poison upon the living particles which are suspended in the atmosphere. The agents which I myself believe to be the most efficient are sulphurous acid, iodine, and carbolic acid. Further experience will probably point to many others, but we have these at present to rely upon.

A few words as to the methods of practically employing these various agents:

1. *Sulphurous Acid.*—If the chamber to be disinfected is (*a*) *uninhabited*, all articles capable of being bleached should be removed; orifices communicating with the external air should be closed, and crevices pasted over with paper. A vessel containing burning sulphur should then be introduced, and the door closed. For the sake of safety from the chance of fire by the overflow of the molten sulphur, it should be kindled upon an iron plate, and floated upon a large vessel containing water. It is convenient to place glowing embers on the plate, and then dust over them flowers of sulphur. If the room is (*b*) *inhabited*, the sulphurous acid must be disengaged in less proportion. Dr. Hjaltelin, who has employed it in small-pox wards, considers that its power of exciting bronchial irritation has been exaggerated, and that patients may be accustomed to it in a high degree. It may be employed in the manner before described; the flame of the burning sulphur being extinguished by submersion of the iron plate in the water, if the fumes become too powerful. Or, as employed by Dr. A. W. Foot, flowers of sulphur may be dropped upon a heated shovel, and carried about the room. The generation of the gas should be repeated three or four times in the day. It is certain, however, that some persons feel it difficult to tolerate the sulphur fumes.

2. *Iodine* may be used as an air-disinfectant in a very simple manner—first suggested, I believe, by Dr. B. W. Richardson. Solid iodine is merely exposed in glass or porcelain vessels in different parts of the room. The iodine vapor is given off at ordinary temperatures. This is a very efficient mode of attaining a constant disinfection.

3. *Carbolic Acid.*—To employ this agent when the room is (*a*) *unoccupied*—communication with the external air being closed—a quantity of the crude acid should be poured into a strongly heated cup, or upon bricks, and left to vaporize. Subsequently, the floors, ceilings, and, if practicable, the walls, should be irrigated or sprayed with an aqueous solution; and the floor scrubbed with carbolic acid or coal-tar soap. Or Savory & Moore's excellent vaporizer may be employed, whereby large volumes of carbolic acid vapor may be disengaged. If the chamber is (*b*) *occupied* by the sick, the vaporizer just mentioned, which is so constructed that the liquefied acid falls, drop by drop, upon a heated plate, should be put in action at intervals two or three times in the day. The objectionable odor of the carbolic acid may be greatly masked by its admixture with oil of wild thyme. It is obvious that this method gives off the carbolic acid too powerfully for constant use. The agent, however, is so volatile as to render itself readily available for maintaining a constant antiseptic atmosphere. It has frequently been used in combination with inert powders for strewing about the room, or has been sprinkled upon cloth or other absorbent materials which have afforded surfaces for its volatilization. These measures, however, have their inconveniences and even dangers; for the strong acid is irritant and caustic, and may inflict serious injury upon those who inadvertently touch it. To obviate the difficulty, I have requested Messrs. Savory & Moore to construct for me an instrument consisting of a piece of canvas in the form of an endless band stretched between two rollers, and dipping into a tin trough to be charged with the acid. By turning a handle at the top, the roller is drawn through the trough, and thus moistened through its whole extent. After it has become partially dry from evaporation, a turn of the handle lifts up a fresh moistened surface. I have used this in several cases, and have found it to act most satisfactorily. I believe, from the data we possess, that in carbolic acid we have the most efficient and most manageable of all agents for the disinfection of air.

